# Medical Miscellanies

**Published:** 1816-11

**Authors:** 


### PART IV.

### MEDICAL MISCELLANIES.

Quicquid agunt Homines.

### MORBID ANATOMY.

(Selected and abridged from Morgagni,)

WITH COMMENTARIES.

(Continued from page 167.)

"Wherever Morbid Anatomy has been steadily adhered to as the foundation of observation and reasoning in our science, that science has been progressive: it has been interrupted in its course, even amidst the most brilliant reputation of its professors, wherever men have arisen, whose inventive powers have led them to reject, rather than to seek the aid of Anatomy."—*C. Bell, on the Diseases of the Urethra.*

*Letter IV. Serous Apoplexy.*

Although Morgagni does not rank himself among those who, "when they find a little water within the skull of an apoplectic person, immediately conclude, that this was the cause of the disorder." Yet, he thinks there is good reason for the division of apoplexies into sanguineous and serous, as the various histories from Valsalva and himself will shew.

Case 1. *from Valsalva.* Valereo Zani's father died of apoplexy; his uncle of stone in the bladder. He himself evinced a gross habit of body; flaccid muscles; short, fleshy neck; rubicund face. He led a sedentary, literary life, indulging in rich food. At the age of 41, he became troubled with stone, and discharged many small calculi; at the same time, he had a copious discharge of saltish saliva from the mouth, with carious teeth. This discharge ceased before his 61st year, and was succeeded by occasional pain and heaviness in the head. About the 63d year, after a rigid observance of Lent, he complained of violent pain and difficulty of making water, which returned periodically, with a month's interval, lasting several days each period. These symptoms were accompanied with heavy pains in the head, especially after any mental emotion; to which succeeded dulness of intellect, with a debility of motion on the right side. As autumn approached, his legs swelled and became œdematous; the skin of the right burst, and discharged a great quantity of liquid serum, which coagulated by heat. In the winter solstice, and during the prevalence of the south winds, he was found, one day, speechless; his right side almost immoveable. After a momentary renovation from this state, he sunk into a kind of apoplectic lethargy, which in five days terminated in death.

*Sectio Cadaveris. Abdomen.* Stomach turgid with air; kidnies soft, but sound; bladder sound, but containing a rough, reddish, oval stone the size of a pullet's egg. *Thorax.* Lungs sound; heart large, and in the left ventricle an incipient polypous concretion. *Cranium.* Dura mater apparently corrugated; under the pia mater, within the sulci of the cerebrum, a limpid stagnant serum. There was more of this in the right than left hemisphere. Two ounces of a similar serum, but of a saltish taste, were found in the larger ventricles. The plexus choroides within the right ventricle contained a vesicle the size of a filbert. In the left, were some small vesicles. Finally, The carotid and vertebral arteries shewed every where, on their internal coats, marks of incipient ossification.

REMARKS. The observations of Valsalva and Morgagni on this case, contain nothing interesting to the pathologist of the present day. The case itself is otherwise. The long established flow of saliva from the mouth, on the principles of a modern physician of the first eminence [Dr. Parry] prevented, or at least, kept in check, the determination of blood to the brain. From the cessation of this discharge, the cerebral congestion was evinced by the head-ache, &c. and from this period may probably be dated the origin of the vesicles in the plexus choroides. The effusion of water in the ventricles, &c. appears the last effort of Nature to procrastinate, even for a few days, the existence of the patient. The effusion into the cellular membrane of the lower extremities warded off, for a time, this final catastrophe.

CASE 2. A professor of law at Bologna, ætat. 60, whose face was of a red, inclining to a leaden colour, had complained about a month, of weakness and pain in the inferior part of the chest, which forced him to rest frequently when he walked even the shortest distance. While sitting in the great church of St. Petronis, he suddenly fell down with a confused kind of scream, and a peculiar contortion of the body. His face was very livid; he foamed at the mouth; discharged the fæces involuntarily; became quite motionless, and died in less than an hour. Although soon after death, his face turned pale, yet a livor was perceptible behind the ears, and in other parts of the body for twenty-four hours.

*Sectio Cadaveris.* Some serum escaped while removing the brain out of the cranium. The sanguiferous vessels of the encephalon were not very turgid; but at the sides of some of them was a small quantity of coagulated lymph; pia mater easily separated; the whole cerebrum extremely flaccid, and of a bleached appearance. In the lateral ventricles, a very little serum of a saltish taste. In the thorax, the lungs appeared as if dyed with ink, but did not adhere to the pleura costalis. In pericardio, a very little water; heart very flaccid; spicks of ossification in the arch of the aorta. The abdomen was not examined. The apoplexy is attributed by Morgagni to the *salt serum*, and to the flaccidity of the heart. In all probability abdominal examination would here have shewed visceral derangement in the neighbourhood of the epigastrium.

CASE 3. A slender man, ætat. 40, laboured under acute fever. In the night of the ninth day, loss of speech; incapable of understanding any sign; very little sensation or motion in his limbs; face not red. Died on the thirteenth day.

*Sectio Cadaveris.* Stagnant serum between the meninges; ventricles full of water.

REMARKS. There can be no doubt that turgescence, if not inflammatory congestion of the vascular system of the brain terminated here in effusion; an event that happens in fevers oftener than is probably imagined by Brunonian practitioners.

CASE 4. A Sexagenarian, of a sallow complexion, had long been afflicted with ulcers in his legs; these being nearly dried up, he was seized with aphonia; stupefaction; and general hebetude. The day following, he died.

*Sectio Cadaveris.* Water was effused into the ventricles of the brain, and between the meninges. For explanation of this case, *Vide* Parry.

CASE 5. A citizen of Bologna, ætat. 70, of a pallid countenance, had difficulty of hearing; occasional vertigo, and faintness; a tremor, attributed to the handling of quicksilver. In other respects, healthy, robust, indulging freely in venereal enjoyments with a young wife. After walking abroad with a friend in unusually good spirits, he was found dead in the road an hour after their parting.

*Sectio Cadaveris.* Abdomen not opened; thoracic viscera sound. The mouth drawn to one side; brain soft, flaccid, and discoloured; serum between the meninges, and in the ventricles; on the left vertebral artery, ossified spots.

Case 6. The Prelate Baptista Anguissola, of large stature, ruddy complexion, and subject to disorders of the urinary organs, *having dried up an old ulcer of his leg*, was seized in the 61st year of his age, first with a fainting fit as he was rowed in his gondola at Venice; soon afterwards, he fell down in his own house without any apparent cause; and last of all he was attacked with apolexy, and declared by the physicians to be at the point of death. Nevertheless, he lived from the middle of July to the middle of August, during which time he took various purging medicines; was bled from the foot, the arm, the hand, and the forehead. Blisters were applied to the skin; cupping-glasses to the crown of the head, &c. but speech could not be restored, nor motion to the right side, which was paralytic. In the mean time, a putrid fever supervened, for which bark was administered, and thoracic inflammation was suspected. At length stertor came on, and he died.

*Sectio Cadaveris.* The convex surface of the liver was marked with long lines of a red colour inclining to brown. Three or four calculi in the gall-bladder; other abdominal viscera sound; urinary bladder thickened in its coats; thoracic viscera healthy. Pericranium red in some places; water flowed from the cavity of the cranium while it was sawing through; vessels on the surface of the cerebrum and cerebellum more turgid than usual; an ash-coloured jelly here and there between the pia mater and convolutions of the brain. Substance of the brain rather flaccid. In the ventricles a little water.

Remarks. This is another instance of the danger of drying up long-established drains, without substituting others in their stead, or obviating the consequences by abstemiousness and evacuations. No person can doubt that this was a *sanguineous* determination to the head, ending in serous effusion. What was denominated putrid fever, was probably no other than *phrenitis*, which the bark aggravated, while it occasioned those symptoms that were suspected to be indicative of thoracic inflammation!

Case 7. A young Venetian, ætat. 29, deformed, and an *inebriate*, staggered, fell down in the street, and immediately expired, in the month of October 1707. His face was livid, and the wine he had drunk, flowed from his mouth and nose mixed with blood.

*Sectio Cadaveris*, by Morgagni and Santorini. Liver and spleen enlarged, the former hard and whitish; the latter soft. Pancreas indurated. The other abdominal viscera a good deal displaced in consequence of a distortion of the spine, which was curved to the left side seven inches from a right line, the aorta following the unnatural curvature. The bony parietes of the chest so much distorted, that the basis of the heart, which was rather large, came close to the lower part of the neck. In the right ventricle, a small polypous concretion; lungs, trachea, and larynx sound. The vessels of the pia mater, especially on the right and inferior side of the brain, were turgid, with very grumous and coagulated blood. Under the pia mater, in several places, among the convolutions of the brain, was found serum. In the plexus choroides were vesicles full of water. Cerebrum rather firm.

Remarks. The propinquity of the heart to the head; the obstruction to the free flow of blood through the obliquities of the aorta; the indurated liver retarding the return of blood from the cœliac and mesenteric arteries; the state of inebriety, and the heat of the season, sufficiently elucidate the *post mortem* appearances.

Case 8. An ostler, near 60 years of age, tall, fat, and accustomed to eat much and drink freely, was brought into the hospital at Padua in the middle of December 1725. This was the fourth time of his admission here. 1st. He had been received for a short, and not violent fever. 2dly. For an apoplexy, from which he recovered. 3dly. For an inflammation in his throat, accompanied by incontinence of urine;—and, finally, for apoplexy again, which terminated fatally in ten hours after he was found, lying in the blanket with which he had wrapped himself three days before. When discovered, he was paralytic in the right side, and speechless.

*Sectio Cadaveris.* On opening the abdomen, the bladder presented itself distended with urine and reaching the navel; it was covered with fat, as were the kidnies and ureters. Its coats were thickened, and its fundus in a state of incipient ulceration. The kidnies were unequal on the external surface; their pelves were enlarged; their glandular substance extenuated. The right kidney had a cell partly prominent and partly hollowed out from its body, full of a fluid-like urine. The ureters were greatly enlarged, especially the right one, which was tortuous, and exceeded in size the aorta; it measured more than thirty fingers' breadth in length! It was a little contracted as it approached the bladder, but greatly dilated, like a funnel, where it joined the kidney. The stomach was extremely small, and in the middle of its posterior and external surface was a roundish tubercle, apparently similar in colour and substance to the stomach itself. On raising the sternum, the lungs were seen shrunk up on each side to the back, so as to leave a very fat mediastinum altogether uncovered. The whole surface of the heart adhered closely to the pericardium. The carotid arteries, as they ascended the neck were unusually large. The cerebrum was remarkably firm, the cerebellum not so. There was a considerable quantity of water both between the meninges and in the ventricles of the brain. The vessels of the pia mater turgid. The posterior branches of the carotids within the head exceedingly dilated. Ossified points were found in the left of these branches, and also in the left vertebral, near its anastamosis, which lessened greatly their diameter.

Case 9. N. Ferrasina, a Veronese Priest, formerly supposed to be consumptive, at Venice, and who had laboured under hemicrania ten years before at Padua, was now in the 43d year of his age. His hair was grey; his face sometimes flushed; his habit of body slender. In temper apparently sprightly; but in reality, very anxious with dissembled cares, and very prone to anger. He complained often of pains in the thorax, the seat of which he pointed out by laying his hand on the sternum. One evening, in the month of May, the season being very warm, he supped cheerfully, but not immoderately, with his friends; and early the next morning was found dead in the posture of sleep, and without any foam at his mouth.

*Sectio Cadaveris.* The dura mater at the sagittal suture was black with blood. The vessels of the pia mater turgid with the same. The medullary substance of the brain was brown, as was supposed from the quantity of blood, as the sanguiferous vessels appeared through its substance. In the lateral ventricles, and also in the tube of the cervical vertebræ was a pretty large quantity of water. No disorder was apparent in any other part of the brain or its coverings. The lungs were sound, but containing much blood. In the pericardium, no moisture. In the right ventricle, a polypous concretion. The carneæ columnæ of the left ventricle inflamed. The semi-lunar valves in a state of incipient induration. The arch of the aorta, from the heart to the descending portion, had an unequal surface externally, like knots in a tree: internally, there was only a wrinkled surface, with two small marks of incipient ossification. In the abdomen, no appearance of disease.

Remarks. The pain within the thorax doubtless proceeded from the aortic lesion, perhaps from the chronic inflammation of the carneæ columnæ. Did the "dissembled cares" and proneness to anger, arise from the cardiac lesion, or the *latter* from them? The temper seems greatly influenced by cardiac, as well as hepatic diseases; and on the other hand, the organ of circulation, and that of biliary secretion, suffer much from mental emotions. Whether any connection existed between the cardiac lesion and cerebral congestion and effusion here, it may be difficult to determine.

Case 10. A rustic, near Bologna, 60 years of age, had long been afflicted with foul *ulcers in his legs,* which he was very anxious to have healed up, though he was of a bad habit of body, and so irregular in his bowels as seldom to have a stool oftener than one in six days without medicine. At length, having met with "*a very officious surgeon,*" the matter was so far brought to a conclusion, that in three months the ulcers were nearly healed. The cicatrix, however, was not completed, when he, one day, complained suddenly of his head. On the third day, he began to be delirious, and presently lost the sense of feeling all over his body. At length stertor came on, and he died.

*Sectio Cadaveris.* Hard excrements were found distending the colon and other large intestines in several places. The liver was distinguished by very small spots of a tawny colour, variegated like marble; and this viscus, which was rather larger than usual, had a disagreeable smell. The gall bladder was contracted, and nearly void of bile. The spleen very large, and covered with oblong spots of a black colour. Nothing remarkable in the thorax. When the cervical vertebræ were divided from the thoracic, water flowed out from their tube. In the skull, a thin fluid was found betwixt the dura and pia mater; and betwixt the latter and the brain.

Remarks. Morgagni, from the *manner* in which he relates this case, evidently conceives the healing up of the ulcer to have contributed to the fatal catastrophe which ensued, though he does not expressly say so. At all events, the case is worthy of attention.

Case 11. An octogenarian, who had formerly been afflicted with ulcers in the legs, as the scars evidently shewed, was admitted into the hospital in the evening; his skin was almost covered with filthy pustules. His pulse was weak and unequal; less sensibility in one arm than in the other. His eyes glistened, and were fixed; yet seemed to look at certain objects. Being asked if his head was painful, heavy, or drowsy, he answered, No. But he said, faulteringly, that he had vomited. His sense, feeling, and motion remained. He died next morning.

*Sectio Cadaveris.* The *liver whitish and somewhat hard;* its bladder filled with black bile. A stricture in the colon, just below the stomach. *Considerable quantity of water in the brain.*

Here finishes Morgagni's Letter on *serous* apoplexy. He seems to be of opinion, that the effused water is sometimes the *cause* of apoplexy by its pressure, and sometimes only the *effect* of that turgescence of the vessels, which of itself can induce the disease. Can modern Pathologists controvert these positions, or add any thing new to them?

( To be continued. )

---

*Case and Dissection by a Physician.*—The subject of the accompanying examination, was a delicate married female, little more than twenty years of age. She had borne one child, and was advanced about four months in pregnancy when I was, on the 17th of March 1814, requested to visit her with another physician. The account the lady gave of herself, was this—That about four years previous to our visit, she had suffered much from what was considered by her medical attendants an inflammatory sore throat; that she was relieved by a quantity of matter being spontaneously discharged from the throat, with a smart fit of coughing. She recovered the power of deglutition, but a cough supervened with loss of voice, and constant large expectoration of frothy mucus and yellow matter. From these symptoms she had never been free; though the cough and expectoration were less at intervals; but they were constantly aggravated by an accession of cold. She further stated, that the physicians she had consulted were not unanimous in their opinions; some considering her case as consumption and some not.

On enquiry we found, that she had never suffered from pain in any part of the thorax; she could make a deep inspiration with perfect ease, and but for the constant irritation of the fauces compelling her to cough, could lye in any position. She breathed quick and short; but this she attributed to the incessant cough, and indeed I did not conceive it arose from any affection of the lungs. There was no particular perspiration, though the hectic flush was constant in the evening. Her pulse was 100. The bowels were habitually costive; and the appetite was much impaired. After the first visit in consultation, the patient was consigned solely to my care. The chief remedies employed till the 28th of March, were opiates, gentle laxatives, and low diet. After that period, as the cough and spitting were much abated, I ventured on gentle tonics with an opiate at night, combined with some blue pill; and desired the diet might be improved. This plan was continued till the 8th of April: when, in consequence of slight ptyalism, the blue pill was omitted, and the power of the tonic increased. She had now nearly lost her cough and spitting. Her appetite was good; the hectic flush had disappeared; her voice was improved, and her breathing free; she continued to progress favourably, and on the 16th, I considered her so far recovered as not to require medical aid. On the 17th, having passed a comfortable day, she retired to bed in good spirits at ten o'clock, and slept till half past twelve; when she awoke with an exclamation, that she was dying, and, jumping out of bed, sunk lifeless upon it.

On Tuesday, April the 19th 1814, about thirty-three hours after death, the body of the deceased was examined. Conceiving from the symptoms which had occurred during her sickness, that the seat of disease was in the trachea, that part was first inspected; there were several patches of puriform matter in its course; but no very evident traces of recent inflammation were observable.

Just within the larynx was seen a soft fimbriated, or wart-like excrescence, about the bulk of a middle-sized hazel nut. This excrescence was firmly and broadly attached to the edge of a sac or sinus, formed behind the mucous membrane, and which sinus extended upward and outward into the cellular substance, about an inch. The extent of what may be called "the mouth" of this sinus was about half an inch, and its edges were thickened: there was no opening from it through the larynx.

To this sinus and excrescence at the mouth of the larynx may be justly attributed, it is conceived, not only the coughing and exspuition, but also the loss of voice. The origin of the excrescence is assignable, it is thought, to previous ulceration; and the sac or sinus to a former abscess.

On exposing the contents of the thorax, the surface of the lungs, exhibited a healthful appearance; they were attached both o the pleura and pericardium, by numerous strong bands. The substance of the lungs, on minute examination, was found to be perfectly sound. *In the thorax were found about eight ounces of a sanguineous fluid.*

The pleura presented some traces of inflammation. The heart was in a healthy state. There were about six drachms of fluid in the pericardium. In the lower venter, nothing like disease was detectable.

As there was never during the illness of the deceased, any symptom of effusion in the thorax; it is concieved, that the fluid found there must have been suddenly effused, and thus have caused her death.

PATHOLOGY. *Malformation of the Heart* *—On examination of the heart of a criminal, aged 26, the following malformation was presented. The superior vena cava consists of two very distinct trunks, which open separately into the cavity of the right auricle. One of these trunks, the direction of which is vertical, and which occupies the ordinary site of the vena cava, receives only the right subclavian and corresponding external and internal jugular veins; and opens into the superior part of the right auricle. This trunk is the smallest. The other, larger, receives the left subclavian vein and jugulars of the same side, the inferior thyrodeal, left intercostal, and several other branches. This trunk is at first horizontal, but becomes vertical as it crosses before the arch of the aorta and left division of the pulmonary artery: it then passes above the appendix of the left auricle: at the posterior part of which it becomes again horizontal, and enters the left part of the right auricle by an orifice destitute of valves. This orifice is situated to the left of the inferior vena cava, and behind the fossa ovalis which separates it from the opening of the other superior vena cava; so that these two veins enter the auricle at the distance of more than an inch from each other. The cardiac veins open by three distinct orifices destitute of valves, into the horizontal portion of the second trunk, behind the left auricle and not into its cavity by a single orifice as commonly happens. The heart presented no other malformation.

*Remarkable Case of Croup in its greatest state of intensity.*—At a meeting of the Royal Society at Gottingen on the 14th of October, 1815, Professor Blumenbach communicated the following remarkable cure of croup, effected by Dr. Reddelin of Wismar.—Dr. R. insinuated, by means of a quill, a mixture of Spanish snuff and marocco into the nostrils of a girl, 19 years of age, who had been taken with the croup, and who on the third day was already unable to swallow, had begun to rattle in the throat, and appeared approaching her dissolution. This operation being repeated, she, under sneezing and vomiting, brought up two long membranous cylinders; upon which the rattling immediately ceased, and the patient was rescued from instantaneons suffocation. One of the tubes brought up measured, when slit open and extended, nine French lines in breadth; they were quite white, elastic, and bore a strong extension, without injury to their fibrous texture.

---

* *Vice de conformation du cœur. Cas pathologique presenté par M Beclard et Cloquet. Bulletin de la Faculté de Medecine de Paris, &c.* 1816. *No.* v.

*Cow-pock, and Small pox.* In the Grand Dukedom of Baden, 19,018 children have been vaccinated during the year 1814, the 37th of whom was inoculated with spurious matter; and in 41 the inoculation did not take at all. In the same year, 386 children were infected with the natural small pox; of which 75, consequently every 4th child, died. The small pock miasma proved as fatal in that year, as it did heretofore; and 4,500 children were thus rescued by vaccination in one year only in that Dukedom.

In the cabinet of natural and artificial curiosities at St. Petersburgh, are at present 120 embryos, including progressive specimens from the first moment after conception, up to the full growth of the fœtus of nine months. The very first exhibits a mere gelatinous vesicle, that was taken from a woman who had been caught by her husband in the act of adultery, and had been killed by him on the spot.

On Wednesday, October 2, 1816, the Society for Relief of Widows and Orphans of Medical Men in London and its Vicinity, (under the Patronage of his Royal Highness the Duke of Kent,) held their half-yearly General Court, when the following gentlemen were elected into office for the ensuing year:

PRESIDENT, Dr. BAILLIE.

VICE-PRESIDENTS,

| | | |
|---|---|---|
| Sir Francis Milman | Sir William Blizard | Mr. Rendall |
| Dr. Haworth | Mr. Heaviside | Mr. Haworth |
| Sir Gilbert Blane | Mr. Ware | Mr. Ridout |
| Dr. Dennison | Mr. Norris | Mr. Simons |

TREASURERS,—Dr. JOHN SIMS, Mr. UPTON, Mr. STEELE.

DIRECTORS,

| *Physicians,* | *Surgeons,* | *Apothecaries,* |
|---|---|---|
| Dr. Merriman | Mr. Robert Keate | Mr. Atkinson |
| Dr. Outram | Mr. Edward Browne | Mr. Seaton |
| Dr. Maton | Mr. Vincent | Mr. Malim |
| Dr. Warren | Mr. Cartwright | Mr. Wells |
| Dr. Frampton | Mr. Copeland | Mr. Tegart |
| Dr. Farre | Mr. Burrows | Mr. John Simpson |
| Dr. Adams | Mr. Charles Clarke | Mr. Wheeler |
| Dr. Laird | Mr. And. Matthias | Mr. Hayton |

SECRETARY, Mr. William Chamberlayne, Aylesbury Street.

COLLECTOR, Mr. Hunt, St. Martin's Court, Ludgate Hill.

This Society was instituted in the year 1788. Its capital is now Twenty Three Thousand Six Hundred Pounds Three per Cent. Consolidated Bank Annuities, and Two Hundred Pounds Navy Five per Cents.; out of the interest of which, down to the 18th of September, in the present year, the sum of Five Thousand Two Hundred and Eighty Two Pounds has been distributed among the Widows and Orphans of deceased Medical Men, Members of this Society, many of whose families have been left without any provision whatever.

*Insanity.*—We are happy to see, from the last Number of the MEDICAL REPOSITORY, that Dr. Burrows is directing his attention to the pathology of Insanity. We have read his first essay with much interest, and are free to confess that his ideas coincide with those which we long ago formed on the subject. We confidently predict, that the farther the investigation proceeds, the more fully will it be proved, that the *epigastric viscera* are more frequently the centre from whence radiate the various shades of mental derangement, than the brain itself. Dr. Burrows has zeal and ability to pursue the enquiry; and we hope, that through the liberal encouragement of the profession, he will be supplied with materials. We hope also, that the Asylum over which he presides will afford ample scope for his talents, and meet the approbation and support of his brethren.

Mr. HOWSHIP has in the Press, "Practical Observations in Surgery and Morbid Anatomy, illustrated by Cases; with Dissections and Engravings."

MR. THOMAS JOSEPH PETTIGREW, F. L. S. &c. &c. has in the Press, "Memoirs of the Life and Writings of the late JOHN COAKLEY LETTSOM, M. & LL. D. F. R. S. F. A. S. F. L. S. &c. &c. &c. with a Selection from his Correspondence with the principal Literati of this and Foreign Countries."—The two first volumes will consist of a Memoir of Dr. LETTSOM, drawn from original and authentic sources; and of a selection from his very extensive correspondence with numerous distinguished characters.—In order to render this department of the work as perfect as possible, the Editor respectfully solicits the communication of such facts, documents, and letters, as may be in the possession of any of Dr. LETTSOM's friends; which will be not only thankfully recieved, but faithfully acknowledged.—The remaining volume will contain the medical correspondence, and a collection of cases, papers, &c. These will be illustrated by engravings.

DEATHS.—At Croom's Hill, Greenwich, James Hurdis, M. D.—At Hydrabad, John Campbell, Esq. Surgeon in the East India Company's Service on the Madras Establishment.—In Hanover Square, Dr. S. H. Jackson, aged 61.

---

